# Supplementary material for: A long non-coding RNA Leat1 mediates the hormone responsiveness of EfnB2 during male urogenital development
Source: Commun Biol. 2025 Dec 15;9:57. doi: 10.1038/s42003-025-09322-y (PMC12800023; doi:10.1038/s42003-025-09322-y)
Supplement: Supplementary file 2 — Supplemental Information [file 42003_2025_9322_MOESM2_ESM.pdf]

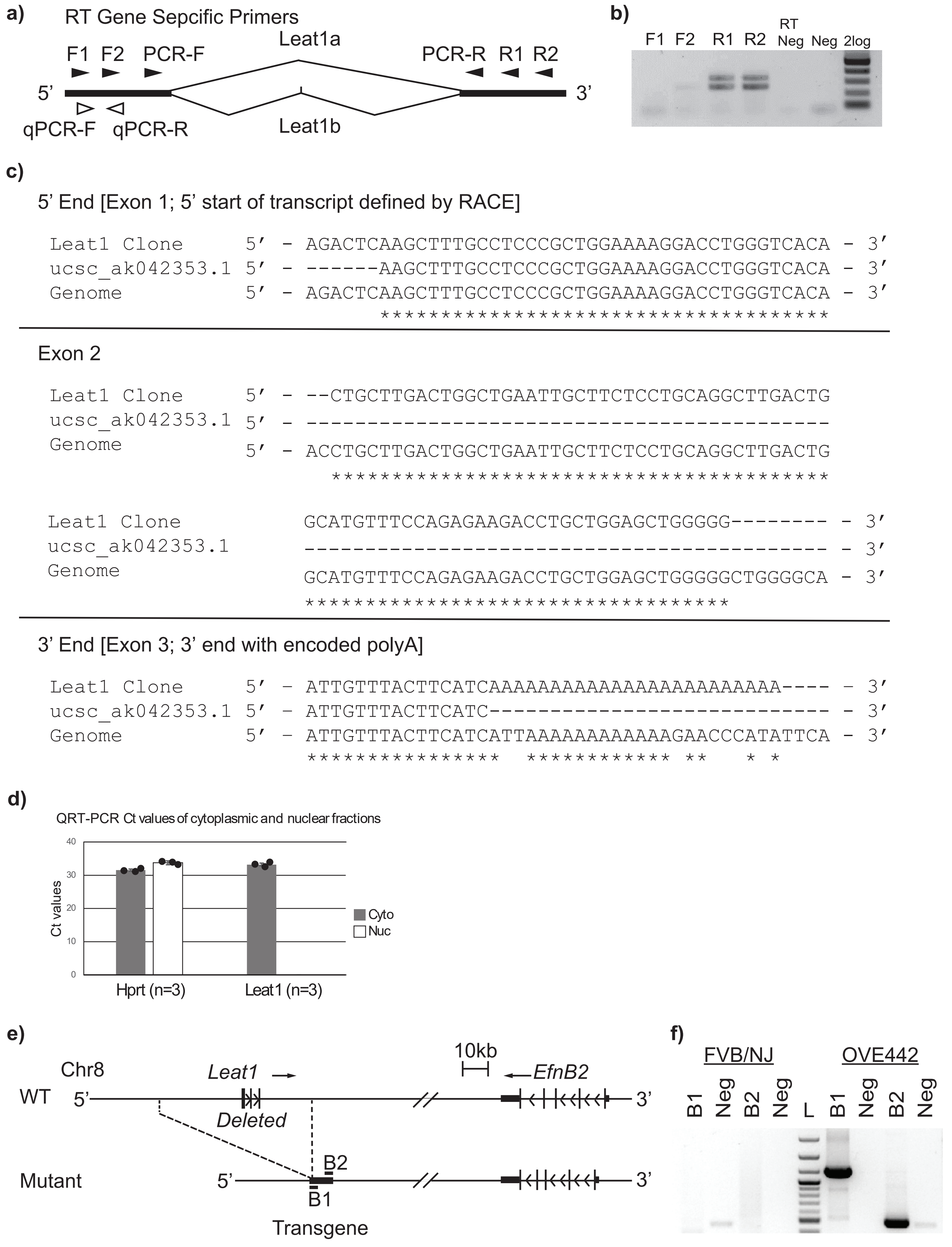
a)
RT Gene Sepcific Primers
F1 F2 PCR-F
Leat1a
PCR-R R1 R2
5'
3'
qPCR-F qPCR-R
Leat1b
b)
F1 F2 R1 R2 RT Neg Neg 2log
c)
5' End [Exon 1; 5' start of transcript defined by RACE]
Leat1 Clone 5' - AGACTCAAGCTTTGCCTCCCGCTGGAAAAGGACCTGGGTCACA - 3'
ucsc_ak042353.1 5' - ------AAGCTTTGCCTCCCGCTGGAAAAGGACCTGGGTCACA - 3'
Genome 5' - AGACTCAAGCTTTGCCTCCCGCTGGAAAAGGACCTGGGTCACA - 3'
****
Exon 2
Leat1 Clone 5' - --CTGCTTGACTGGCTGAATTGCTTCTCCTGCAGGCTTGACTG
ucsc_ak042353.1 5' - ---
Genome 5' - ACCTGCTTGACTGGCTGAATTGCTTCTCCTGCAGGCTTGACTG
****
Leat1 Clone GCATGTTTCCAGAGAAGACCTGCTGGAGCTGGGGG-------- - 3'
ucsc_ak042353.1 --- - 3'
Genome GCATGTTTCCAGAGAAGACCTGCTGGAGCTGGGGGCTGGGGCA - 3'
****
3' End [Exon 3; 3' end with encoded polyA]
Leat1 Clone 5' - ATTGTTTACTTCATCAAAAAAAAAAAAAAAAAAAAAAAA---- - 3'
ucsc_ak042353.1 5' - ATTGTTTACTTCATC---------------------------- - 3'
Genome 5' - ATTGTTTACTTCATCATTAAAAAAAAAAAAGAACCCATATTCA - 3'
*************** ************ ** * *
d)
QRT-PCR Ct values of cytoplasmic and nuclear fractions
Ct values
40
30
20
10
0
Cyto
Nuc
Hprt (n=3)
Leat1 (n=3)
e)
Chr8
Leat1
10kb
EfnB2
WT
5'
3'
Deleted
B2
Mutant
5'
3'
B1
Transgene
f)
FVB/NJ
OVE442
B1 Neg B2 Neg L B1 Neg B2 Neg

Supplemental Fig 1. Characterization of *Leat1* transcript. **a)** Schematic representation of *Leat1* transcripts. Black arrowheads represent *Leat1* primers used in this study. F1, F2, R1, R2 were used to determine the orientation of the RNA in b). PCR-R and PCR-F represent primers used to detect *Leat1* in nuclear and cytoplasmic fractions. qPCR-F and qPCR-R show positions of primers used in the qPCR experiments. **b)** Agarose gel electrophoresis of RT-PCR products from amplification using primer sets depicted in a) showing that *Leat1* is transcribed exclusively from the plus strand of genomic DNA. 2log shows the 2log DNA ladder, RT Neg shows PCR on native RNA, Neg shows template minus control. **c)** Sequence of the *Leat1* clone, obtained after RACE PCR (*Leat1* Clone) showing alignment to the genome (Genome) and cDNA clone from NCBI database (ucsc_ak042353.1) (https://www.ncbi.nlm.nih.gov/nuccore/AK042353). Exon 2 sequence is not present in clone ucsc_ak042353.1. We defined an additional 6bp of open reading frame at the 5' end of the transcript compared with ucsc_ak042353.1 and a polyA tail encoded in the genome and present in the transcript at the 3' end. Panels D-G show whole mount in situ hybridization of *Leat1* in the male GT at E13.5. **d)** Ct values from quantitative RT-PCR for a housekeeping gene *Hprt* and *Leat1* performed on cytoplasmic and nuclear fractions derived from E14.5 male GTs (n=3 biological replicates). Although *Hprt* was amplified from both fractions, *Leat1* could only be amplified from the cytoplasmic fraction. **e)** Schematic representation of the transgene insertion in the mutant mice (Mutant) compared to wild type. The *Leat1* mutation resulted in a loss of approximately 50kb of genomic DNA on chromosome 8 with at least 2 copies of transgene insertion. The genomic rearrangement was located approximately 300kb downstream of the *EfnB2* termination sequence. Within the deleted region, only *Leat1* was entirely removed. **f)** Confirmation of location of transgene insertion and genomic deletion by PCR. B1 and B2 primers were designed at the boundary of the transgene insertion. The FVB/NJ strain, the parent strain of *Leat1* mice, was used as negative control. The deletion interval was detected only in *Leat1* mutant mice but not FVB/NJ. Neg, no DNA control; L, 1kb ladder.

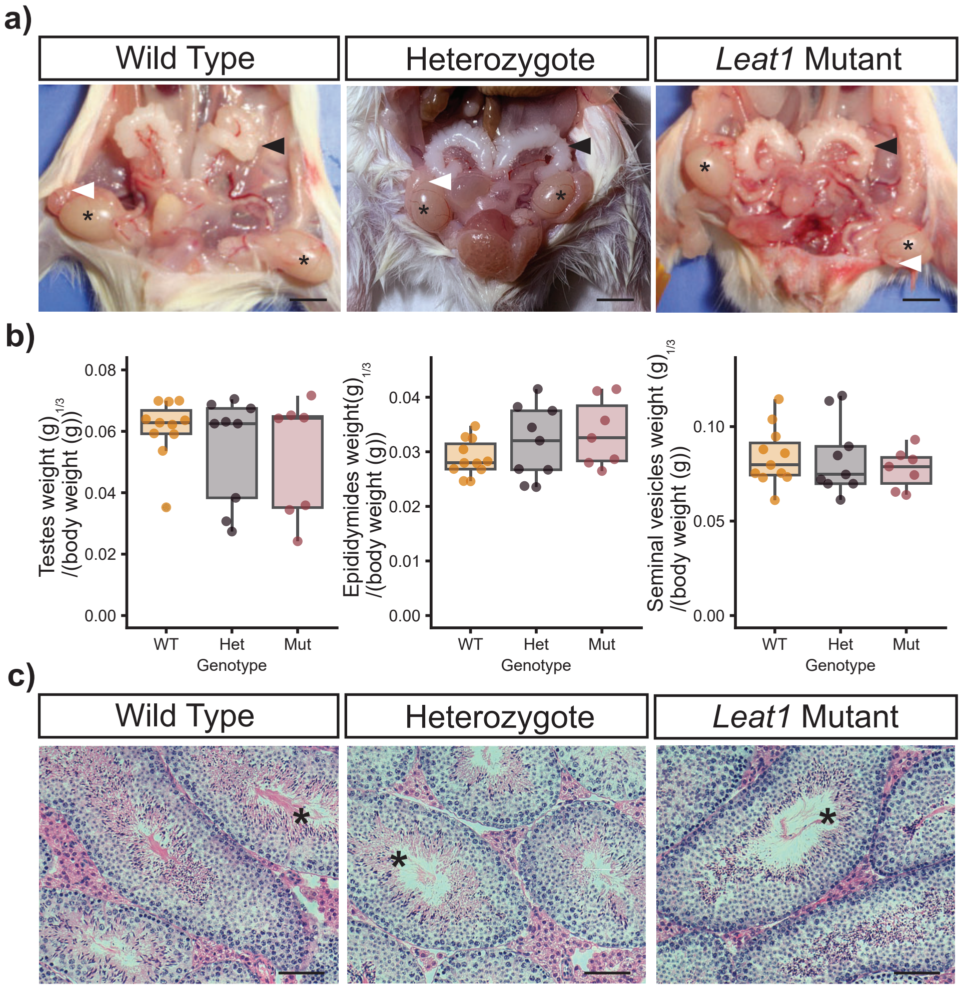

a)
Wild Type
Heterozygote
Leat1 Mutant
b)
Testes weight (g)1/3 /(body weight (g))
0.00
0.02
0.04
0.06
0.08
WT
Het
Mut
Genotype
Epididymides weight(g)1/3 /(body weight (g))
0.00
0.01
0.02
0.03
0.04
WT
Het
Mut
Genotype
Seminal vesicles weight (g)1/3 /(body weight (g))
0.00
0.05
0.10
WT
Het
Mut
Genotype
c)
Wild Type
Heterozygote
Leat1 Mutant

Supplemental Fig 2. Gross morphology of the internal reproductive tract of wild type, heterozygote and *Leat1* mutant adult males. **a)** The highly androgen sensitive seminal vesicles (black arrowhead), epididymides (white arrowhead) and overall testis size (*) were similar between wild type and mutant mice indicating normal virilization. Scale bars = 0.5 cm. **b)** Testis, epidymides and seminal vesicle weights are not significantly different between wild-type (n=11), heterozygote (n=9) and *Leat1* mutant (n=7) adult males. **c)** Representative transverse testis sections reveal normal spermatogenesis in wild-type, heterozygote and *Leat* mutant adult males, with mature spermatid (*) present inside testicular tubules. Scale bars = 50 μm.

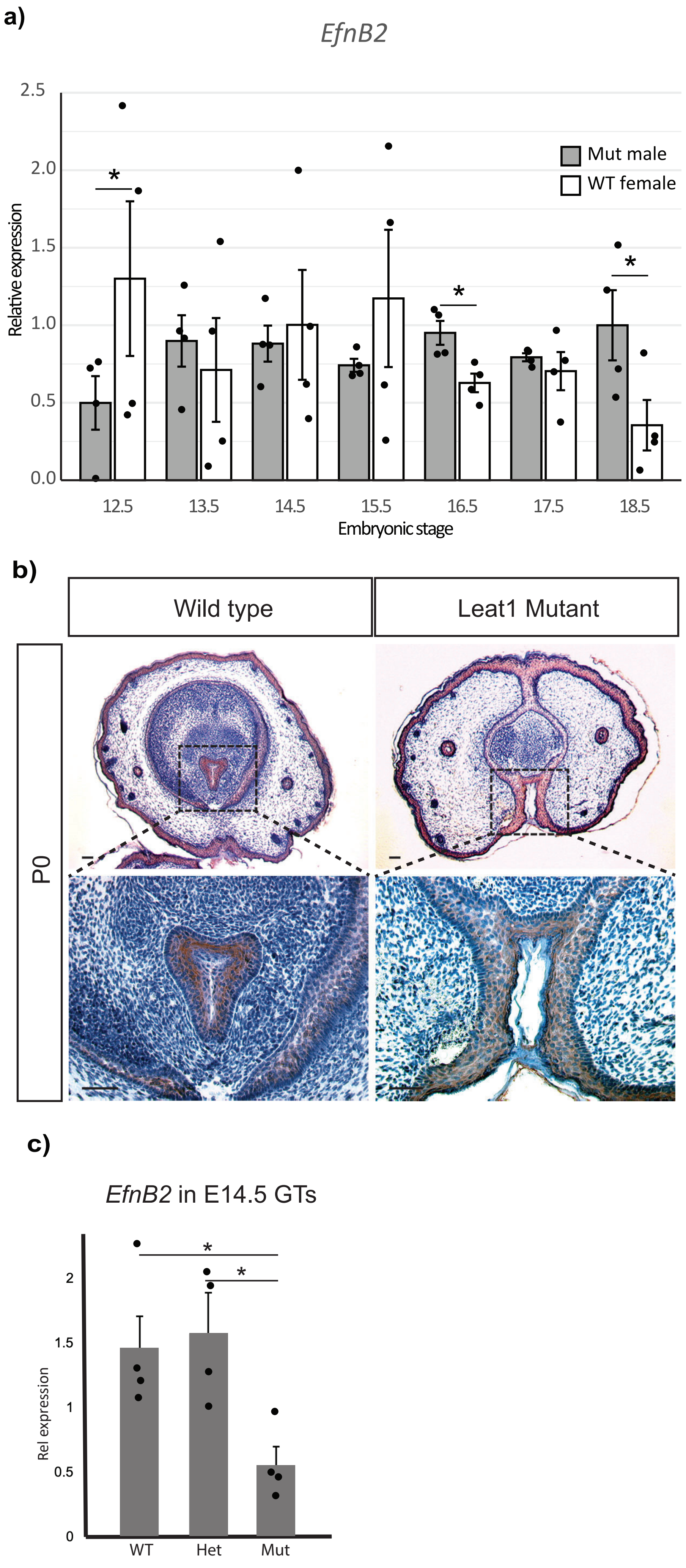
a)
EfnB2
2.5
2.0
1.5
1.0
0.5
0.0
Relative expression
Mut male
WT female
*
*
*
12.5
13.5
14.5
15.5
16.5
17.5
18.5
Embryonic stage
b)
Wild type
Leat1 Mutant
P0
c)
EfnB2 in E14.5 GTs
Rel expression
2
1.5
1
0.5
0
*
*
WT
Het
Mut

Supplemental Fig 3. *Leat1* regulation of *EfnB2*. **a)** Quantitative real-time RT-PCR showing relative expression of *EfnB2* (mean ± standard error of mean (SEM)) in the male mutant (black bars, n=4 at each stage) and female wild type (white bars, n=4 at each stage) genital tubercle throughout embryonic development. *EfnB2* in the *Leat1* homozygous male GT is significantly reduced compared to female WT GTs at E12.5 and is not significantly different at E13.5, E14.5, E15.5 and E17.5. The variability observed in this data reflects the biological heterogeneity of long non-coding RNAs. *EfnB2* expression is relative to expression in E12.5 WT male genital tubercles. * = $p<0.05$. **b)** EPHRINB2 is similarly distributed in the GT at P0 in both wild type and *Leat1* mutant males. EPHRINB2 is located in the urethra epithelium and in the epithelium of the urethral folds, which are not fused in the *Leat1* mutant. Scale bars = 100µm. **c)** *EfnB2* (mean ± standard error of mean (SEM)) is not significantly reduced in male *Leat1* heterozygote (n=4) genital tubercles at E14.5 compared to wild type male genital tubercles (n=4). Both wild type and *Leat1* heterozygote male GTs have significantly higher *EfnB2* than *Leat1* mutant GTs (n=4). Expression was detected by quantitataive RT-PCR and expressed as relative to expression in E12.5 WT male genital tubercles. * = $p<0.05$.

Alignment quality color range: BAD AVG GOOD

Leat1 Alignment Exon 1

```
Mouse    AGACTCAAGCTTTGCCTCCCGCTGGAAAAGGACCTGGGTCACAGTGTCTGAGTCTGTATACGCTGCCACCAA
Human    GCC-------------------------------T--------------------G----------------
Wallaby  GAAT--------------------------------------------------------------------
cons

Mouse    AGGCTGACTTACACAATTGTTCTGATCGCAGTTTTGAACCTGATGTCTGCGCCACCTTACCTGCCGCATACT
Human    -------------------TTC-------A------------------------------------------
Wallaby  ------------------------------------------------------------------------
cons

Mouse    TTGAAGACTAGGGTTGCACCTTCTGCGAGGGCAGAGGAATCCTGTCACTGACAGACCACCTTCGAATCCCTC
Human    ----------G-------------------GCA-----A---------------------------------
Wallaby  ------------------------------------------------------------------------
cons

Mouse    GCTTGGTGAAGTGTGACTTCTAAGTGCTAGACCAGCTTCTAAGAACAGCCTACTTTCTACTTGCAGCTGAAA
Human    -----------T--GAC---------------------------------TACTT-----------------
Wallaby  ------------------------------------------------------------------------
cons

Mouse    ACGAAGGTTGTGTGCTATCGTGAATCGGATATTAGTCGTTAACGAGACGCTCCTTGATAGGAGGTGGCTGTC
Human    -------------------------------TTAGG-------------------------------C----
Wallaby  ------------------------------------------------------------------------
cons

Mouse    CTTGGCCATGTGCTTCCTGAAGCCTTGAAGAAAAGCCACAGCATTTCCTGCTGGTGCCGCTCCTGGGGTGGC
Human    -------A----------G-----------------------------------------------------
Wallaby  ------------------------------------------------------------------------
cons

Mouse    ATCACCAGGCTTTGTTGCACCTCATACTGGATACGTGACCACGGTTGCCAGCTTCTCTCTCCGTGTCATTAT
Human    ------------------------------------------------------------------------
Wallaby  ------------------------------------------------------------------------
cons

Mouse    CATGGAACCTACATGCTGTGACCACTGTGATTTCTGACCGGCTATCACTCAGCCTGCTCAGGCATTGACTAT
Human    ------------------------------------------------------------------------
Wallaby  ------------------------------------------------------------------------
cons

Mouse    TTTTAGGCAGAAGGTCTAGTCCCCGCTTTTATTCCCACAGCCGGCTTCTATTTTAATGCTCCATTTGGCTCC
Human    ----------AGGTTCTAGTCCCTAGTTTTATTCCCAGAGCCGTCTTCTATTTTAATGCTCCATTTTGCTCT
Wallaby  -----------------------------------------------------TAAT---------------
cons                                                              ****
```

```
Mouse    AGGAACCAAACCATAGCCGTGGAATTCCCACAGGGCGTTAACAGAAAGCAAAACCTCCTCCCACTTTGGAAT
Human    AAGAACCGAACCATGCCCATAGAAGTCCCACAGGATTTTTGTAGAAAGGCAAACTTCCTCCCACGCCGGAAT
Wallaby  -------GAACCATCC--------------------------------------------------------
cons             ******
```

```
Mouse    TAATGAACCCCTCTCGCTGTCAGCCATGCTTTAATGGATTTCACCAGTCTCTGGATTTCCACACCCAGAACA
Human    TAATGAGCCCCTCTTGCTGTCAACTGTAGTTTAATGGATTTCACCATTCTGTGTATTTTCACACCCACAACA
Wallaby  -------------CTGCTGTCAGCTCTGCTTTAATGGGTTTCACCATTCTGTATATTTTCATACCCAGAACA
cons                    ******* *  *  ******** ******** *** *  **** ** ***** ****
```

```
Mouse    GA-AAGTCATACAAAG
Human    GA-GAGTCATACAAAG
Wallaby  GAAAAGTCATAC--AA
cons     **  ********  *
```

Leat1 Alignment Exon 2

```
Mouse    CCCCCAGCTCCAGC--AGGTCTTCTCTGGAAACATGCCAGTCAAGCCTGCAGGAG-AAGCAATTCAGCCAGTCAA
Human    GTGCATATTTCAAGGGAAGAAGTTTCAAAAAATGAAAAATGAAGGCC-TAGGAGGGTACAGATTATGAAG--GA
cons        *    * **    * *   * **   ***      * * *** *   *****    **  * *   **   *
```

```
Mouse    GCAG
Human    TGAA
cons       *
```

Leat1 Alignment Exon 3

```
Mouse    CAAACACC---ACCA---TGAAAGG---ATGAAGAGA---TAGGATATGCAAAAGGAGG-AGATGTTGGCAGTGA
Human    CAAAGATATGGAACAAGCCCAAATGCCCATCAATCAACAAGTGGATAAAGAAATTGTGGTAAATATATATCATGG
cons     **** *     * **     *** *   ** **   *     *****   ***  * ** * ** *      **
```

```
Mouse    CGTGTTTCTAACTTGTTAACAAGACTGCAGTA--------TCTATCAGCTTCGG-GAGCAGGGCACGAGGTTGGG
Human    AATACTACTCAGCCATAAAAAGGAATTAAATAATGGCATTTGCAGCAGCCTGGAAGTGGAGACCATTACTCTACG
cons       *  * ** *    * ** * ** *  * **        *  * **** * *  * * **  **  *   *  *
```

```
Mouse    AG--------GAAGAATGGT-GGCAAGATTCTGAAAGCTACTGGGCTATTTGT-TGAGATAAA-AATGGGGATGG
Human    TGAAGTAATTCAGGAATGGAAAACCAAACATTGTATGTT-CTCGCTCATAAGTGAGAGCTAAGATATGAGGATGC
cons      *         * ******    * * *   ** * * * ** *   **  **  *** ***   *** *****
```

```
Mouse    GCATCTCCAAACTT--CCCTTTATCCTTCCTTGGATGAAGAAAAATTAA-AGTTAATTTGTGTTCCTTAAAGAAA
Human    AAAG--ACAAGAATGATACAATGGACTTTGGGGAGTCAGGTGAAAGGGTGAG-AGAGGGGTGAGGGATAAAAGAC
cons       *    ***   *    *  *   ***    *  * * *  ***     **   *   ***     ****  *
```

```
Mouse    CACAATTGTTGGGGA-AATGTGGTCCCCTCAC--AAAGGAAAAGCC---ATCTAAGCTAATAGCATCAAT-----
Human    TACA--CGTTGGGTACAGTGTACACTGCTTAGCTGATGGGTCCACCAAATCTCAGAATCACCACTAAAGAACT
cons      ***   ****** * * ***   *  ** *    * **     **   **** **  *  * **  **
```

```
Mouse    -TTTCATCACAGTGATT-GG-ATTCTTACCTAAGAA---------ACGGCCCTCTG-GGTTCCTTCAGGGAAGAA
Human    TATTCATGTAACCAAACACCACCTGTTTCCCAAAAACCTATTGAAATTAAAAAATAATAATAATAAAAAGAAAAA
cons       *****   *   *        * ** ** ** **         *        *     *  *  *  *** **

Mouse    TGTTCTT---TCATAAAA--AGACCCA---GGAAAAGAACTTGCCATGAAACCCCAGGATTATCAGCCACACCAT
Human    TATTATTATCTAATAAAAAGAGACCCACAGGGTGAGGAACTTGCCATCCCATCCCAGAAACATCCCTTCCTGTAC
cons     * ** **   * ******  *******   **  * ***********   * ***** *  ***     *   *

Mouse    GTCACAGTGGCTTACT-CTACT---GAC-------TTAC---CTCATGCAAGTGTCACCTGCCACGGTGAGACCA
Human    ATCCCAATGATTTTCAGCCATTAGATACGAATATCAGAGTTTATGATGTTTGCATCAACTGCCATTTTAAGACCA
cons      ** ** **  ** *  * * *    **         *     * ***   *  *** ******   * ******

Mouse    CAGAGGGAAGACTGGCAGGTGGATCTGCACCCTACAGTGATCCATCCTTCAGAAGGATGACTG-----TGGCTTC
Human    ---AGAGAAGACTGAAAGAGAAGTCAGAAACCTGAAGCCATCAA-ACTGAAGATGAATAGCTGGTGAATAGCTGC
cons        ** ********  **     ** * * ***  **  *** *  **  *** * **  ***     * *** *

Mouse    -CTCCTCC---AGTCAGGAAATAGTGTGTGGTCTATTGATCACGCCTCTGCTGTTCAGG-CTCATGACTAGAGGC
Human    CCTTCTCTTTATGTGAAGAAA-AGTAAGCTG-CCGTTGTCTACATTTCAATTAATCAAGTATTATTACTTGTAGT
cons      ** ***     ** * **** ***  *  * *  ***   **   **   *  *** *  * ** *** *  *

Mouse    TGGGAATGCCTGAAGTGATACACAGACATTCTAAACATGAAAGCAGGCATCACACCTGAGAAAATGGCCTGATAC
Human    TAAAAGCAGTTTAACAAATACACAAACCTTCTAGATACAAAAACAAAACTCACCCCTCAGGGGTCAGCTCCAGA-
cons     *   *     * **   ******* ** ***** * *  *** **    **** *** **      **   * *

Mouse    TTCTAAGGGATCAGCAACACTGCTGGTTGCCTTGGTGAGAACAGTGAGGAAACAGGACATCACCTCCTACACTGG
Human    TTCTAGAGGATTTGTCACACAGCTGGTCACCTTGGTGGGACCAGTGAAGAAATGGGATGCAATCTCTTATACCGA
cons     *****  ****  *  **** ******  ******** ** ****** ****  ***    * *** ** ** *

Mouse    AGTCCCTC--CTGCCCCTGTGACTGTCCTAGT-----AAC-TAG-----------CAAGAAGT-----AAAGTGT
Human    AGTTCCTTCACTGCCTCCATCACTGTTCTAAAGAGACAACATAGTAAACTCAAAGCAATACATTCTTAAAAGTAT
cons     *** ***   ***** *  * ***** ***       *** ***           *** *  *     ***** *

Mouse    CG---TATATAAAAC---AAG-ACATGAATTGTAGCAAGATCAAACCCTCCGTGATCGGGTTATGGTTGCATTTC
Human    TATTTCATGTAAATTTATATGTAGATGAATTACAACAAGATGAAACCCTTGGTGGTGGGTTAGTGGTTGCATTTA
cons           ** ****     * * * *******  * ****** *******  *** * ** *  ***********

Mouse    ACAAGA--TTGCTTGACTTCATTTCTAATTTGTTGAATTCCCAGCATATACTACTCTTATAATCAGAAAATAACA
Human    ATTTTCTTTTGCCATTGTCCGTTTTTAATTTATTGAATTTTCAGCGTATGCTACTTTCACAATCAGAAAATGA--
cons     *       ****     * * *** ****** *******  **** *** ***** * * *********** *

Mouse    CAAATATGACTTCTAGAGTGAAAAAAAATGTAGTTCCCACATTATCATTTCAACACTAATCAAGAACCAAAAATA
Human    CAAATATTATCTATAAA----ATAAAAGTATAGCTTCTGCCTTATCATTTCAACACCAATCAAGAACCAAAAGC-
cons     ******* *  * ** *    * **** * *** * *  * *************** ***************

Mouse    AAGGTGGCGCCGGGCAGCAGACCTTAAATGAGAGGCTGTTTTCCCAAGCTCTGGTTAAGATAGGAATGTGAGATA
Human    AAGGTGGCACCAGTCAGCAAGCCTTAAATTAAAGGGTGTTTTCCCAGTTTCTGGTTAAGATAGGAATGCAAGACA
```

```
cons   ******** ** * *****  ******** * *** **********   *******************  *** *

Mouse  CAGCTGAGGTGACATGTGTGAAT-CACCCAGCATGTTCCCAACAACCACAGCTTTAAAGCAACATCCCTCATTCT
Human  CAGTAAAGGTGACAGGTATGTATTTACACAGCACAGTCCCAACAAC-----------------------------
cons   ***   ******** ** ** **  ** *****   **********

Mouse  CTCCCCTGCTTTGTGTAATTCTACATACAAAGCTGTCTTTCTGGCTTCTTCTCTATCCCGTTATCCCTAATAGTT
Human  ----------------------------AA-----T---------------------------------------
cons                               **     *

Mouse  CCACAGATTCTATATAAGGGCTATTAATACACTAAAAGGACAAAATTGTTTACTTCATCA
Human  -CATAG--------------CT-T--------T-AAAG-----------------C--AA
cons    ** **              ** *        * ****                 *   *
```

Supplemental Fig 4. *Leat1* nucleotide sequence is conserved across species. Multiple sequence alignment for Mouse, Human and Wallaby *Leat1* nucleotide sequences were performed using T-COFFEE [59].

Supplemental Table 1. Primers used for the study.

Supplementary Table 1

mLeat1 cloning primers	Clm353F, Forward 5'- GTACTAGCTAGCAAGCTTTGCCTCCCGCTGGAAAAGG -3'
	Clm353R, Reverse 5'- ATAAGAATGCGGCCGCGATGAAGTAAACAATTTTGTCC -3'
mEfnB2 CDS cloning primers	ClmEfnB2F, Forward 5'- CACCATGGCCATGGCCCGGTC -3'
	ClmEfnB2, Reverse 5'- GACCTTGTAGTAAATGTTGGCAGGACTC -3'
mEfnB2 in situ probe	ClimEfnB2F, Forward 5'- CAAATGGGTCTTTGGAGGGC -3'
	ClimEfnB2R, Reverse 5'- CCCAGGGTCGGAAAAGCTAC -3'
Genotyping OVE primers mutant	OVEMutFwd, Forward 5'- CCACATTTGTAGAGGTTTTACTT -3'
	OVEMutRev, Reverse 5'- CCCTGGTATCACTGAACAATCA -3'
Genotyping OVE primers wild type	OVEWTFwd, Forward 5'- GACATGCCTGTTCATATCTTGGC -3'
	OVEWTRev, Reverse 5'- CTTTGAGGCTCTTTGCATTGTGA -3'
Transgene insertion Genomic boundary 5'	B1F, Forward 5'- AGGAATTGGGGTTAATGGTTGGT -3'
	B1R, Reverse 5'- AGCCATACCACATTTGTAGAGGTT -3'
Transgene insertion Genomic boundary 3'	B2F, Forward 5'- CCACATTTGTAGAGGTTTTACTTGC -3'
	B2R, Reverse 5'- CCCTGGTATCACTGAACAATCA -3'
Leat1 RNA orientation RT-PCR	OuterRT353F1, Forward 5'- TGCTCAGGCATTGACTATTT -3'
	InnerRT353R1, Reverse 5'- ATGCCCATCCCCATTTTTAT -3'
	InnerRT353 F2, Forward 5'- GTCTAGTCCCCGCTTTTATT -3'
	OuterRT353 R2, Reverse 5'- TCCAAGGAAGGATAAAGGGA -3'
	V5EfnB2, Forward 5'- CAAGGTCAAGGGCAATTCTGCAGATATCCAG -3'

Supplementary Table 1

Exogenous EfnB2 qRT-PCR	V5EfnB2, Reverse 5'- TCAATGGTGATGGTGATGATGACCGGTACG -3'
Endogenous EfnB2 qRT-PCR	EndEfnB2, Forward 5'- CAAGGGAACTCGCACCTTGTTCTTGGGCAC -3'
	EndEfnB2, Reverse 5'- GTGGGGATCTCCTAGCAGTCTTCCAGCTTC -3'
Actin qRT-PCR	Actin, Forward 5'- CAACTGGGACGACATGG -3'
	Actin, Reverse 5'- GCAACATAGCACAGCTTCTC -3'
Hprt qRT-PCR	Hprt, Forward 5'- GAGGGTCCTGTTGATGTGCCAG -3'
	Hprt, Reverse 5'- GGTGGCTTAGGCTCATAGTGC -3'
Leat1 qRT-PCR	Leat1, Forward 5'- TCGCAGTTTTGAACCTGATG -3'
	Leat1, Reverse 5'- GCTGGGCTTTTCTTCAAGG -3'
EfnB2 qRT-PCR	EfnB2, Forward 5'- CTCAACTGTGCCAGACCAGA -3'
	EfnB2, Reverse 5'- GGATCCAGGCCCTCCAAAG -3'
Arid1B qRT-PCR	Arid1b, Forward 5'- GCCGCGCAACAAAGGAGTC -3'
	Arid1b, Reverse 5'- AGTAGCCACTCACAGCTTGC-3'
Cux1 qRT-PCR	Cux,1 Forward 5'- GCTGAACACCCTGAAGTCCA -3'
	Cux1, Reverse 5'- CCCTTTTCTCCTGGCTGACC -3'
Frmd4 qRT-PCR	Frmd4, Forward 5'- AGCTCTTCTTCCTGAATGCCAA-3'
	Frmd4, Reverse 5'- GTAGCGCTGGCAGCTTTTTC-3'
Aust2 qRT-PCR	Aust2, Forward 5'- TCACCCCAATATGTTCGCCC-3'
	Aust2, Reverse 5'- CTCTGAGAGGCCAGGAAACG-3'

Supplementary Table 1

Frem2 qRT-PCR	Frem2, Forward 5’- GAATTGGGCGTGGATCTCCTT-3’
	Frem2, Reverse 5’- AGGCTCCGAGAGAACCACTT-3’
Ryr2 qRT-PCR	Ryr2, Forward 5’- AGCTGGAAGACCCTGCAATC-3’
	Ryr2, Reverse 5’- ACCAGGCTGAAATATCCCCG-3’
Sim2 qRT-PCR	Sim2, Forward 5’- TTCCCGGAAGGTCTAGGAGA-3’
	Sim2, Reverse 5’- GCCACCACGAACACAAATCC-3’
Mafb qRT-PCR	Mafb, Forward 5’- TTCGACGTGAAGAAGGAGCC-3’
	Mafb, Reverse 5’- GTAGTTGCTCGCCATCCAGT-3’
