# Supplementary material for: A long non-coding RNA Leat1 mediates the hormone responsiveness of EfnB2 during male urogenital development
Source: Commun Biol. 2025 Dec 15;9:57. doi: 10.1038/s42003-025-09322-y (PMC12800023; doi:10.1038/s42003-025-09322-y)
Supplement: Supplementary file 3 — Description of Additional Supplementary Files [file 42003_2025_9322_MOESM3_ESM.pdf]

Description of Additional Supplementary files

File name: Supplementary Data

Description: The source data behind the graphs in the paper
